# Editorial: AI and machine learning application for neurological disorders and diagnosis

**DOI:** 10.3389/fnhum.2025.1558584

**Published:** 2025-02-11

**Authors:** Kandala N. V. P. S. Rajesh, Venkata Mani Vakamulla, T. Sunil Kumar

**Affiliations:** ^1^School of Electronics Engineering, VIT-AP University, Amaravati, India; ^2^Department of Electronics and Communication Engineering, NIT Warangal, Warangal, India; ^3^Department of Electrical Engineering, Mathematics and Science, University of Gävle, Gävle, Sweden

**Keywords:** brain-related diseases, electroencephalogram, MRI, artificial intelligence, deep learning

Neurological disorders disrupt the normal functioning of the nervous system, which includes the central nervous system and the peripheral nervous system (Cleveland Clinic, 2024). These disorders encompass a wide range of conditions, such as, epilepsy and seizures, dysarthric speech, schizophrenia, attention deficit hyperactivity disorder (ADHD), Parkinson's disease, brain strokes, and Alzheimer's disease, among others (Johns Hopkins Medicine, 2024).

The causes of these disorders vary and can include genetic mutations, congenital abnormalities, infections, or injuries to the nervous system. Diagnosing these conditions often involves various tests, such as electroencephalography (EEG), electromyography (EMG), nerve conduction studies, imaging tests, and sleep studies.

While these diagnostic tools are essential, manually analyzing the results over extended periods can be prone to errors and is both time-consuming and labor-intensive. To address this challenge, computer-aided diagnostic (CAD) systems are increasingly being developed using machine learning (ML) models. These technologies can assist clinicians by providing reliable second opinions, reducing the workload, and improving efficiency by significantly reducing the time required for diagnosis (Rangayyan and Krishnan, 2024). This Research Topic focuses on advancing methodologies for identifying various neurological disorders by applying artificial intelligence (AI) and ML techniques.

This Research Topic has provided several computer aided approaches to recognize brain tumors using AI and ML solutions. Brain tumors can manifest through a range of psychiatric symptoms, sometimes accompanied by neurological symptoms, which can complicate the clinical presentation (Ghandour et al., 2021). Krishna Priya and Karuna conducted an experiment to classify brain tumors from magnetic resonance images (MRI). For this, they have used four pre-trained deep learning (DL) models, namely, VGG-19, VGG-16, ResNet50, and Inception V3. The authors have tested their images on all of these models individually and identified the best method for this approach as VGG-19. They have considered a total of 305 MR images after performing some image augmentation techniques to reduce overfitting. Reddy, Batchu et al. proposed a computer-aided diagnosis (CAD) system for brain tumor identification using novel entropy-based thresholding and non-local texture features. This approach has a series of steps, including contrast enhancement, segmentation, feature extraction, and classification. The authors used the tuned single-scale retinex (TSSR) method to increase the MR image contrast and brightness enhancement; later, the tumor regions were segmented using this maximum entropy thresholding method, and local patterns were exploited as a feature and classified the tumor MR images from healthy using various conventional ML methods. The segmentation here plays a crucial role before the classification. Reddy, Rajesh, et al., in their other work, exploited the DL framework to achieve the same objective by reducing the effect of overfitting. They employed various normalization techniques to initialize the weights into layers to reduce the layers and, thereby, reduce the complexity of the model. Another important aspect of this work is that they have classified various pathological MR images from healthy ones, unlike the binary classification scheme.

Swami, Gramann, et al. conducted a study analyzing the event-related potentials (ERP) in EEG analysis. The ERPs are the ensemble of brain responses to specific stimuli or events. The common problem with the technical support of this ERP extraction is the delay between the original event and its recording; this can alter the ERP interpretation. Therefore, the authors devised a solution, using a photodiode to measure these delays. The accurate analysis of the ERP plays a crucial role in diagnosing various neural disorders such as schizophrenia, ADHD, epilepsy, and so on. Epilepsy is a brain condition where people have sudden, unpredictable seizures. Krishnan et al. proposed a method to detect epilepsy using EEG. They employed a method called Gramian Angular Summation Field (GASF) to convert the one-dimensional EEG to two-dimensional images, and later, they used some advanced time-frequency image analysis methods to acquire essential attributes from the images. Finally, these features are fed to the random forest (RF) classifier to get the quantitative results. This method shows the efficacy of the time-frequency approach on EEG for neurological disease identification. In another work, Swami, Maheshwari, et al. exploited an advanced optimization method called the non-dominated sorting genetic algorithm-II to detect epilepsy from EEG. The system uses mathematical features from signal transformations and energy values, reducing unnecessary data. It finds the best features through evolutionary transfer optimization (ETO) and uses a neural network model to detect seizures from test data.

Rahul et al. systematically reviewed EEG-based schizophrenia detection using ML and DL models. Both methods face challenges, like ensuring high-quality data and dealing with ethical concerns. The review emphasizes the importance of combining different techniques to make schizophrenia diagnosis more accurate and highlights the potential of AI in this area. The study by Sathiya et al. (a) explored various discrete wavelet transform (DWT) settings in schizophrenia detection. They decomposed the EEG into various modes using DWT for various levels and empirically tested the important features extracted from these modes at various levels.

Mental health problems among young people are becoming a significant concern worldwide. Dhariwal et al. used AI to study how lifestyle choices like smoking and drinking and environmental factors like air and noise pollution are connected to mental health issues. The study analyzes data from the Cities Health Initiative dataset and applies advanced AI techniques, including ML and DL, to predict trends in mental disorders. Kulkarni et al. drafted an opinion article on the effect of convolutional neural networks (CNNs) on one of these neurodevelopmental disorders known as ADHD. Sathiya et al. (b) proposed a work to detect ADHD using EEG signals. They employed Gabor filters to decompose the signals into various frequency bands and compute the statistical features from these bands. They have provided a statistical analysis of these extracted features to show their efficacy. Finally, Shabber and Sumesh conducted research on Dysarthria, a neurological speech disorder that weakens the muscles involved in speech production, leading to slow, slurred, and unclear articulation. Early detection of Dysarthria is crucial for timely intervention and treatment. They employed amplitude and frequency modulated (AFM) signal modeling to extract relevant speech features and applied various machine learning algorithms for classification. To ensure diverse and reliable results, they analyzed data from multiple speech Dysarthria databases, including TORGO, universal access (UA) speech, and Parkinson's dataset.

In conclusion, this Research Topic explored various ML-based approaches for diagnosing neurological disorders, including schizophrenia, ADHD, Parkinson's disease, and epilepsy. Additionally, MRI-based techniques for brain tumor detection are also examined.
